# Concentration-Dependent Interfacial Engineering with a ZE-2OMe Co-adsorbent for Enhanced DSSC Performance

**DOI:** 10.1021/acsomega.6c00348

**Published:** 2026-05-06

**Authors:** Necip Ali Tuna, Mesude Zeliha Arkan, Mustafa Can, Adem Mutlu, Cem Tozlu

**Affiliations:** † Department of Materials Science and Engineering, 226844 Izmir Katip Celebi University, Izmir 35620, Türkiye; ‡ 37509 Ege University, Solar Energy Institute, Izmir 35100, Türkiye; § Institute of Chemistry, University of Silesia in Katowice, Szkolna 9, Katowice 40-006, Poland; ∥ Graphene Application and Research Center, 445380 Izmir Katip Celebi University, Cigli, Izmir 35620, Türkiye

## Abstract

Controlling charge transport and recombination processes at the titanium dioxide (TiO_2_)/dye/electrolyte interface is a key strategy for improving the efficiency of dye-sensitized solar cells (DSSCs). This study systematically examines the concentration-dependent effects of a methoxy-functionalized aromatic coadsorbent, *4-[5′-(3,5-dimethoxyphenyl)-2,2’-bithien-5-yl]­benzoic acid* (ZE-2OMe). Low-concentration ZE-2OMe primarily improves the TiO_2_/dye interface by mitigating interfacial loss pathways and passivating surface defect states, thereby enhancing charge collection and suppressing recombination. Optical analyses confirm suppressed nonradiative recombination, reflected by increased photoluminescence (PL) intensity and a time-resolved photoluminescence (TRPL) lifetime that extends from 2.84 ns in the control to 4.87 ns at 0.01 mM. XPS confirms carboxylate anchoring of ZE-2OMe on TiO_2_ and shows S 2p doublets, evidencing robust immobilization of the coadsorbent layer. Notably, the incident-photon-to-current efficiency (IPCE) spectra show no additional contribution in the ZE-2OMe absorption window, and dye loading does not increase upon coadsorbent addition, indicating that the short-circuit current density (*J*_sc_) enhancement is primarily driven by interfacial/electronic regulation rather than complementary light harvesting. Electrochemical impedance spectroscopy (EIS) demonstrates that at 0.01 mM, the reductions in R_1_ = 5.90 Ω, R_2_ = 70.1 Ω, and the series resistance (*R*_s_ = 21.8 Ω) indicate more efficient charge transport and suppressed recombination at the interfaces. The J–V measurements show that the photovoltaic conversion efficiency (PCE) increases from 3.5% (control) to 5.4% at 0.01 mM ZE-2OMe, while higher concentrations lead to reduced performance.

## Graphical abstract

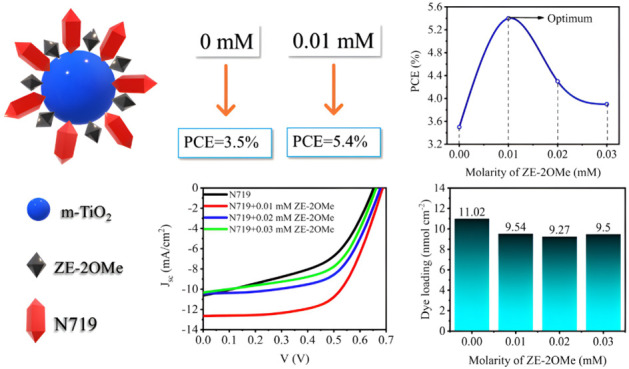

## Introduction

Dye-sensitized solar cells (DSSCs) are third-generation photovoltaic systems in which molecular dyes, chemically anchored to wide-bandgap nanocrystalline titanium dioxide (TiO_2_), harvest sunlight and generate photocarriers.^1–6^ Upon photoexcitation, the dye injects an electron into the TiO_2_ conduction band; the electron percolates through the mesoporous network to the transparent conducting oxide and the external circuit, while the oxidized dye is regenerated by a redox mediator, completing the catalytic cycle. A cascade of steps—light harvesting, electron injection, charge carrier transport, charge carrier collection, and dye regeneration—play key roles for high device performance.^7–9^ Loss pathways, such as intramolecular/intermolecular photodeactivation within the dye layer, back transfer of conduction-band electrons to the oxidized dye, interfacial recombination with oxidized electrolyte species, ionic transport limitations, and parasitic series/shunt resistances, pose obstacles to the efficient realization of photophysical processes.^10,11^ Consequently, precise control of interfacial energetics, defect states, and dipole alignment is crucial to maximizing open-circuit voltage (*V*_oc_), short-circuit current density (*J*_sc_), fill factor (FF), and power conversion efficiency (PCE).^12–14^

High surface coverage of dye molecules on TiO_2_ enhances light harvesting yet often promotes aggregation, which can markedly slow electron-injection kinetics and increase nonradiative losses.^15–17^ Dye aggregation diminishes the electron-injection rate, making the optimization of surface packing density, adsorption geometry (monodentate versus bidentate binding), and overall surface coverage essential to mitigate this trade-off.^16–19^ Coadsorbents provide an effective route to provide this balance by physically suppressing π–π interactions between neighboring dyes and mitigating intermolecular deactivation, leading partially shielding the TiO_2_ surface from electrolyte ions and reducing surface recombination.^18,19^ The magnitude and balance of these functions are highly sensitive to molecular structure and to the coadsorbent/dye ratio; for example, bis-methoxyphenylphosphinic acid (BMPP) typically affords stronger surface passivation, whereas chenodeoxycholic acid (CDCA) more efficiently inhibits aggregation.^19–21^ Beyond steric blocking, competitive adsorption can modulate dye adsorption kinetics, promote bidentate binding, and strengthen dye-surface interactions. Appropriate functional groups can also tune surface dipoles and band alignment, which is reflected in increased *V*_oc_, higher recombination resistance in electrochemical impedance spectroscopy (EIS), and improved fill factor (FF).^22–24^

Passivation of unoccupied TiO_2_ surface sites that remain exposed to the electrolyte after dye adsorption therefore represents a complementary interfacial strategy. Long-chain coadsorbents (e.g., oleic acid) can mask the TiO_2_/electrolyte interface, reducing dark current and suppressing interfacial recombination. Improvements in reduced charge-transfer resistance, extended electron lifetime, and higher PCE have been validated by EIS and Mott–Schottky analyses.^25–27^ Although substantial progress has been achieved using coadsorbents in DSSCs, systematic studies that disentangle the dose-dependent impacts on electron injection, transport, and recombination remain limited, particularly for methoxy-functionalized SAM coadsorbents, where concentration-dependent interfacial behavior has not been explored in sufficient depth. To address these gaps, the present work investigates a dimethoxy-substituted coadsorbent and elucidates its concentration-governed interfacial effects.

The present work systematically maps the concentration-dependent regime of a dimethoxy-functionalized aromatic coadsorbent, *4-[5′-(3,5-dimethoxyphenyl)-2,2’-bithien-5-yl]­benzoic acid* (ZE-ZE-2OMe), introduced in the 0–0.03 mM range in DSSCs. At an optimized low concentration, optical characterizations consistently indicate suppressed nonradiative recombination, reflected by an enhanced photoluminescence (PL) response and a prolonged time-resolved PL (TRPL) relative to the coadsorbent-free sample. In agreement, Fourier Transform Infrared Spectroscopy (FTIR) evidences attenuation of the Ti–OH contribution, while X-ray photoelectron spectroscopy (XPS) confirms carboxylate-mediated anchoring and the formation of a chemically stabilized, passivated interface. EIS analysis further supports improved interfacial charge-transfer kinetics through reduced resistive losses and an extended electron lifetime. These interfacial gains are directly mirrored in the photovoltaic output, yielding simultaneously improved *J*_sc_, *V*_oc_, FF, and overall PCE compared with the untreated device. At higher concentrations, both EIS and J–V parameters degrade, consistent with overcoverage, steric disorder, partial blockage of transport pathways, and renewed recombination.

## Experimental Section

### Materials

Fluorine-doped tin oxide (FTO)-coated substrates were supplied by Kintec. 18NR-AO opaque TiO_2_ paste and the EL-HPE high-performance redox electrolyte were provided from DYESOL. The ruthenium dye, ditetrabutylammonium cis-bis­(isothiocyanato)­bis­(2,2′-bipyridyl-4,4′-dicarboxylato)­ruthenium­(II) (N719), was purchased from DYESOL. An aqueous chloroplatinic acid solution (8 wt % in water; Sigma-Aldrich) was utilized as the platinum precursor for the fabrication of the counter electrode. TCI supplied 5-bromo-5′-(4,4,5,5-tetramethyl-3,2-dioxaborolan-2-yl)-2,2′-bithiophene. [4-(Methoxycarbonyl)­phenyl]­boronic acid, 3,5-dimethoxybenzeneboronic acid, 1,2-dimethoxyethane (DME), and N,N-dimethylformamide (DMF) were purchased from Alfa Aesar. Sigma-Aldrich provided [1,1′-bis­(diphenylphosphino)­ferrocene]­dichloropalladium­(II) as the Pd catalyst, as well as ethanol and dimethyl sulfoxide (DMSO, ≥99.7%). Potassium carbonate (K_2_CO_3_) was acquired from Riedel-de Haën. The synthesis and characterization details of ZE-2OMe are presented in Supporting Information S1–S7.

### Device Fabrication

FTO-coated glasses were cleaned sequentially using a detergent wash and ultrasonic baths (20 min each) in deionized water, acetone, and isopropyl alcohol. The substrates were subjected to an O_2_ plasma treatment for 7 min to further activate the surface. A compact TiO_2_ layer was then deposited via spin coating (2000 rpm, 20 s), followed by thermal annealing at 460 °C for 1 h. Following cooling to ambient temperature, a mesoporous TiO_2_ (m-TiO_2_) film was formed via doctor blading and sintered at 500 °C for 1 h. After annealing, the electrodes were cooled to ∼80 °C and subsequently sensitized by immersion for 24 h in a 0.5 mM N719 dye solution prepared in a DMSO/ethanol mixed solvent (1:4, v/v). For coadsorbent functionalization, a ZE-2OMe stock solution was prepared in DMSO. Separate 3 mL samples were prepared from the N719 dye solution, and ZE-2OMe adsorbent was added to each sample at concentrations of 0.01, 0.02, and 0.03 mM, respectively. Pt counter electrodes were fabricated by dropwise deposition of a platinum precursor solution onto FTO substrates, followed by annealing at 450 °C for 30 min. The effective cell area was defined as 0.25 cm^2^.

### Characterization

Molecular absorption spectra were collected with a Shimadzu UV-3600 UV–Vis–NIR spectrophotometer. N719 dye loading was quantified by desorbing the dye from sensitized TiO_2_ electrodes into 4.0 mL of 0.1 M NaOH solution. The UV–Vis absorbance of the desorbed solutions was recorded using a spectrophotometer with a 1 cm path-length quartz cuvette, and the absorbance at 500 nm was used for quantification. Dye concentration was calculated using the Lambert–Beer law (A = *εcl*, l = 1 cm) with ε(500) = 8176 M^–1^ cm^–1^. Electrochemical impedance spectroscopy (EIS) measurements were carried out on a Zahner IM6 workstation under dark at *V*_oc_ over a frequency window of 100 mHz–1 MHz. The thickness of the m-TiO_2_ layer was determined with an Ambios P7 profilometer. The thicknesses of the m-TiO_2_ and c-TiO_2_ thin films were measured to be approximately 9 μm and 45 nm, respectively (Figure S8). The photoluminescence (PL) and time-resolved photoluminescence (TRPL) spectra of the thin films were acquired using an Edinburgh Instruments system. Current density–voltage (J–V) plots were obtained under simulated sunlight (AM1.5G); the irradiance was set to 100 mW/cm^2^ and calibrated prior to each measurement using a certified silicon control cell (active area: 4 cm^2^). Incident photon-to-current efficiency (IPCE) spectra was measured with an Enlitech QE-R setup. X-ray photoelectron spectroscopy (XPS) measurements were performed using a PHI VersaProbe spectrometer equipped with a monochromatic Al Kα X-ray source (1486.6 eV). The instrument was operated at 24.9 W with a 100 μm beam diameter and a 45° photoelectron takeoff angle, and each acquisition required approximately 1.5 min. Spectral deconvolution and peak fitting were carried out in CasaXPS (v2.3.17PR1.1) employing the LA­(1,643) line-shape function. FTIR spectra were recorded using a Thermo Scientific Nicolet iS50 spectrometer. FTO/TiO_2_ films were immersed in ZE-2OMe stock solution overnight, and analyses were performed on these samples without any annealing treatment. LC-Q-TOF-MS Method: Chromatographic separation was carried out using an HPLC Agilent 1260 Infinity series (Agilent Technologies, Santa Clara, CA, USA) equipped with a binary pump, an online degasser, an autosampler and a Poroshell 120 EC-C18 (3.0 × 100 mm, particle size 2.7 μm) (Agilent Technologies) column was used to separate the compound. The mobile phase system consisted of 0.1% formic acid in water (A) and acetonitrile (B) using a gradient elution as follows: 0–0.5 min, 5% B; 0.5–2 min, 25% B; 2–4 min, 50% B; 4–6 min, 75% B; 6–10 min, 95% B; 10–16 min, 5%B for equilibration of the column. The column temperature was maintained at 35 °C. The injection volume was 10.0 μL, and the flow rate was 0.4 mL/min. MS analysis was performed using an Agilent 6550 iFunnel high-resolution Accurate-Mass QTOF-MS, equipped with an Agilent Dual Jet Stream electrospray ionization (Dual AJS ESI) interface operating in positive ion mode was used at the following conditions: drying gas flow, 14.0 L/min; nebulizer pressure, 35 psi; gas drying temperature, 290 °C; sheath gas temperature, 400 °C; sheath gas flow, nitrogen at 12 L/min. The scan range was from *m*/*z* 50 to 1000, and the acquisition rate was 1.5 spectra/s. The collision energy was 10 eV. Integration and data evaluation were performed using MassHunter Workstation software vffvv (Agilent Technologies, Santa Clara, CA, USA).

## Results and Discussion

To elucidate the effect of ZE-2OMe concentration on interfacial electronics and device performance, structural, optical, chemical, and electrochemical characterizations were performed on DSSC samples prepared with different ZE-2OMe concentrations. The UV–Vis absorption characteristics of ZE-2OMe in chloroform (300–500 nm) are presented in Figure 1a. The spectrum exhibits a narrow, symmetric absorption band in the near-UV, with a maximum at approximately 394–400 nm. Because this band lies outside the characteristic absorption window of N719 (400–600 nm), optical competition and spectral overlap between the two species are minimal. These results indicate that ZE-2OMe functions as an optically passive coadsorbent in DSSCs rather than as a light harvester and thus operates primarily via surface passivation and dipole alignment at the TiO_2_/dye interface.

**Figure 1:**
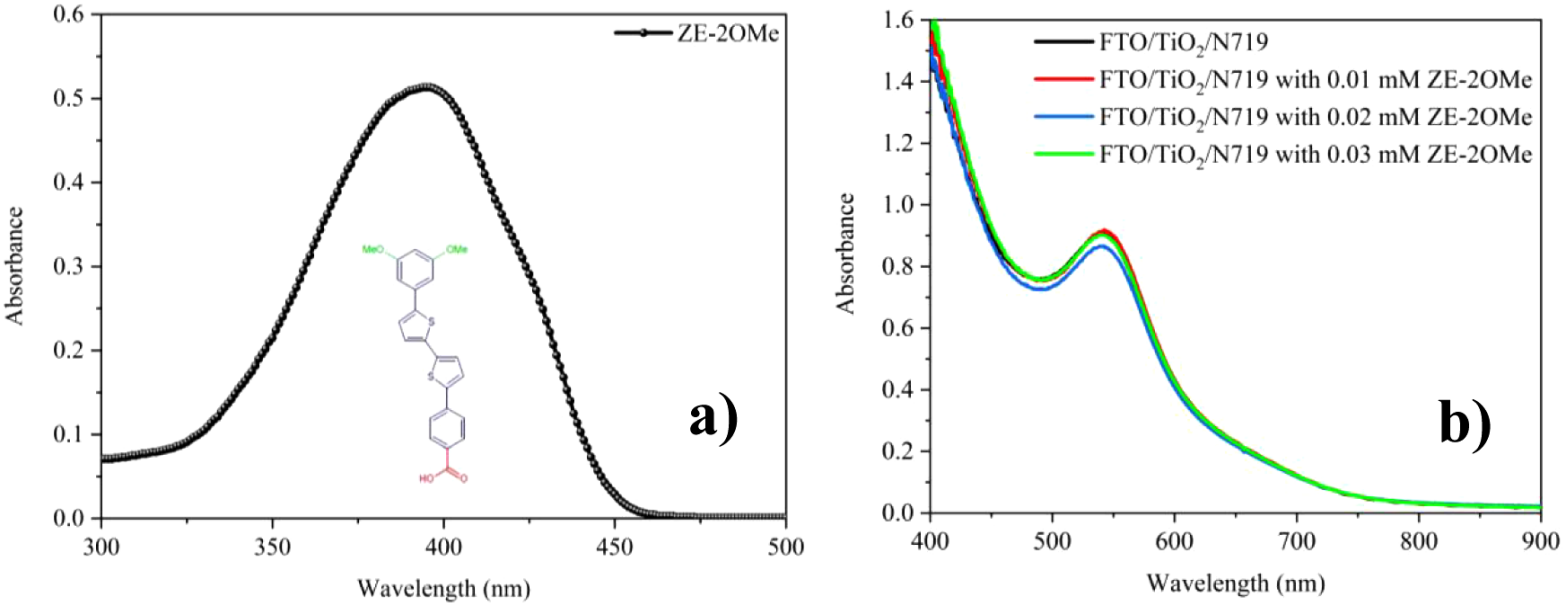
(a) UV–Vis absorption spectrum of the ZE-2OMe coadsorbent in chloroform; (b) UV–Vis absorption spectra of N719-sensitized TiO_2_ thin films recorded after introducing ZE-2OMe at different concentrations.

Figure 1b shows that incorporating ZE-2OMe into the N719 sensitization bath does not measurably alter the optical signature of the adsorbed N719 layer: the spectra largely overlap across the visible region, and the characteristic N719 bands are preserved without any discernible peak shift.^28^ This indicates that ZE-2OMe does not alter the intrinsic electronic transitions of N719 on TiO_2_ and that the overall photon-harvesting capability remains essentially constant within the investigated concentration range. The only notable deviation is a slight reduction in absorbance for the 0.02 mM condition, which most plausibly reflects a modest decrease in effective dye loading caused by competitive adsorption of ZE-2OMe for surface binding sites (or a small change in dye packing density) rather than a change in dye chemistry. Importantly, given the negligible variations in absorbance, the performance gains at low ZE-2OMe concentrations cannot be primarily ascribed to enhanced light harvesting. Rather, they are more plausibly governed by interfacial improvements, most notably strengthened surface passivation and reduced nonradiative losses, as consistently supported by the PL/TRPL and EIS results.^12,29,30^

To clarify whether the performance variation originates from changes in dye loading, the N719 was quantified by desorbing the sensitizer into a 0.1 M NaOH-based solution and analyzing the absorbance at 500 nm.^31^ As shown in Figure S9, the control photoanode exhibits a dye loading of 11.05 nmol cm^–2^, whereas the addition of ZE-2OMe to the dye bath yields slightly lower dye coverages of 9.54, 9.27, and 9.50 nmol cm^–2^ for the 0.01, 0.02, and 0.03 mM conditions, respectively. This corresponds to a modest decrease of approximately 14–16% relative to the control, consistent with partial site competition between the coadsorbent and N719 at higher surface coverage. Importantly, despite this small reduction in dye loading, the optimum ZE-2OMe condition still delivers improved device metrics, indicating that the photovoltaic enhancement is not driven by increased light harvesting from higher dye loading. Instead, the results support an interfacial origin, where ZE-2OMe primarily modulates the TiO_2_/dye/electrolyte interface while maintaining comparable dye coverage.

The PL spectra in Figure 2a compare the photoluminescence intensities of N719 dye containing various amounts of ZE-2OMe. For the TiO_2_/N719 reference, the maximum PL intensity appears in the ∼375–385 nm range. Upon introducing ZE-2OMe, a pronounced decrease in PL intensity is observed. This reduction indicates that the coadsorbent ZE-2OMe passivates surface traps, thereby reducing electron–hole recombination and consequently suppressing PL. In particular, the sample with 0.01 mM ZE-2OMe exhibits the lowest PL intensity, pointing to more efficient charge separation and superior interfacial passivation. The TRPL traces in Figure 2b show how charge-carrier lifetimes evolve with different ZE-2OMe concentrations. Table 1 lists the extracted *τ*_1_, *τ*_2_ and goodness-of-fit values (*χ* ^2^) from the TRPL analysis as a function of coadsorbent concentration. The results demonstrate a clear impact of ZE-2OMe on photocarrier dynamics: at 0.01 mM, the *τ*_2_ lifetime reaches 4.87 ns, its maximum value, consistent with the most effective surface passivation and the lowest level of nonradiative recombination. At higher concentrations, *τ*_2_ decreases compared to 0.01 mM, which can be attributed to partial hindrance of electron-transfer pathways caused by excessive surface coverage.^32^

**Figure 2:**
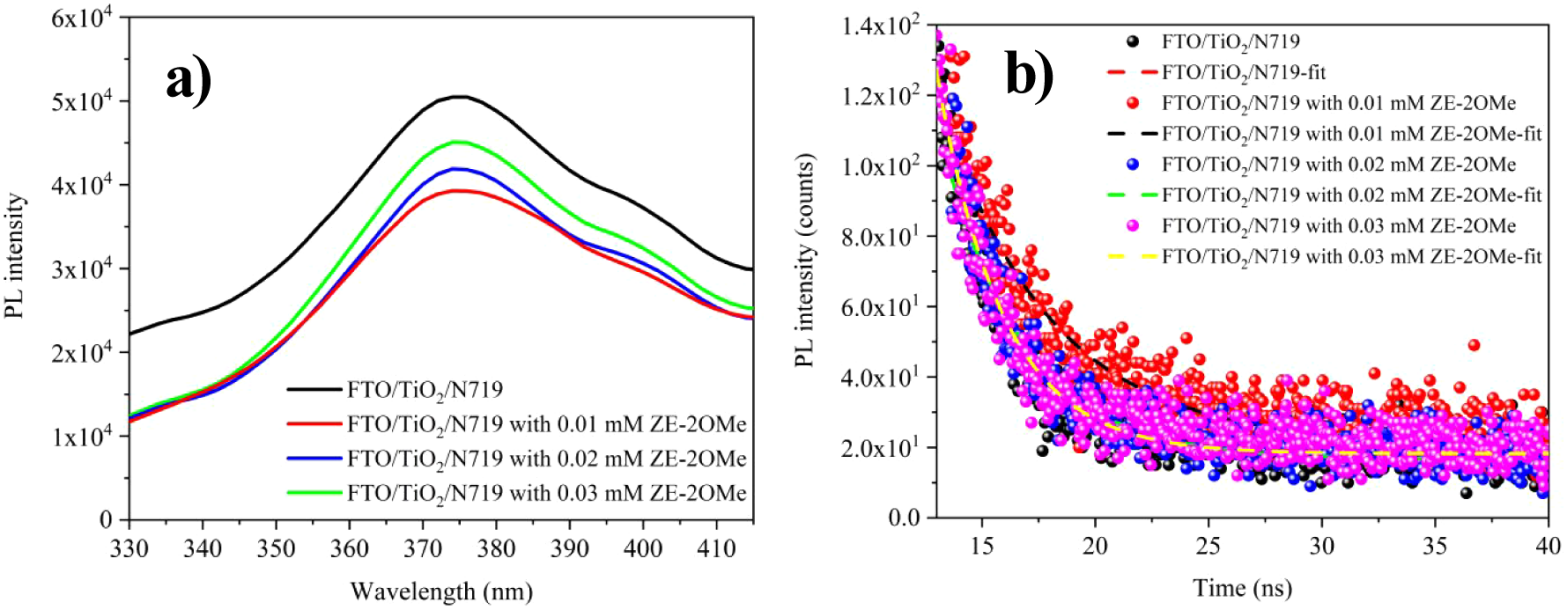
(a) PL spectra and (b) TRPL decays recorded for N719-sensitized TiO_2_ films prepared with varying coadsorbent concentrations, including a coadsorbent-free reference.

**Table 1:** Carrier Lifetime (*τ*) and Goodness-Of-Fit (*χ* ^2^) Values Obtained from TRPL Measurements of N719/ZE-2OMe-Modified TiO_2_ Films as a Function of Co-adsorbent Concentration

ZE-2OMe Conc.	*τ*_1_(ns)	*τ*_2_(ns)	*χ* ^2^
**0**	0.45	2.84	1.192
**0.01 mM**	0.25	4.87	1.304
**0.02 mM**	0.61	3.58	1.258
**0.03** **mM**	0.37	2.85	1.216

The FTIR spectra acquired for TiO_2_/N719 films prepared with different ZE-2OMe amounts show no discernible concentration-dependent changes (Figure S10). Considering the strong and spectrally congested vibrational contribution of the N719 overlayer and potential band overlap, the absence of an apparent loading trend in the coadsorbed films does not necessarily preclude ZE-2OMe adsorption. To directly probe the presence and binding motif of ZE-2OMe, control TiO_2_ films were treated only with ZE-2OMe. Upon ZE-2OMe treatment, the FTIR spectrum of TiO_2_ exhibits clear additional vibrational features attributable to the organic overlayer and carboxylate anchoring. In particular, the pronounced band at 1157 cm^–1^ is consistent with C–O/C–O–C stretching of methoxy/ether groups, supporting the presence of ZE-2OMe on the surface.^33,34^ More importantly, the emergence and strengthening of bands at 1418 cm^–1^ and 1539 cm^–1^ can be assigned to the symmetric and asymmetric stretching modes of surface-bound carboxylate (COO^–^), indicating deprotonation of the carboxylic acid and coordination to TiO_2_.^35^ A new component near 1600 cm^–1^ further suggests the formation of a modified interfacial environment, which may reflect contributions from the conjugated aromatic framework and/or heterogeneous carboxylate binding configurations.^36^ In the 3600–3000 cm^–1^ region, the broad O–H stretching band associated with surface hydroxyls and physisorbed water on TiO_2_ changes noticeably after ZE-2OMe treatment, indicating an altered hydrogen-bonding environment and partial consumption/coverage of surface −OH sites upon molecular adsorption (Figure 3).^37^

**Figure 3:**
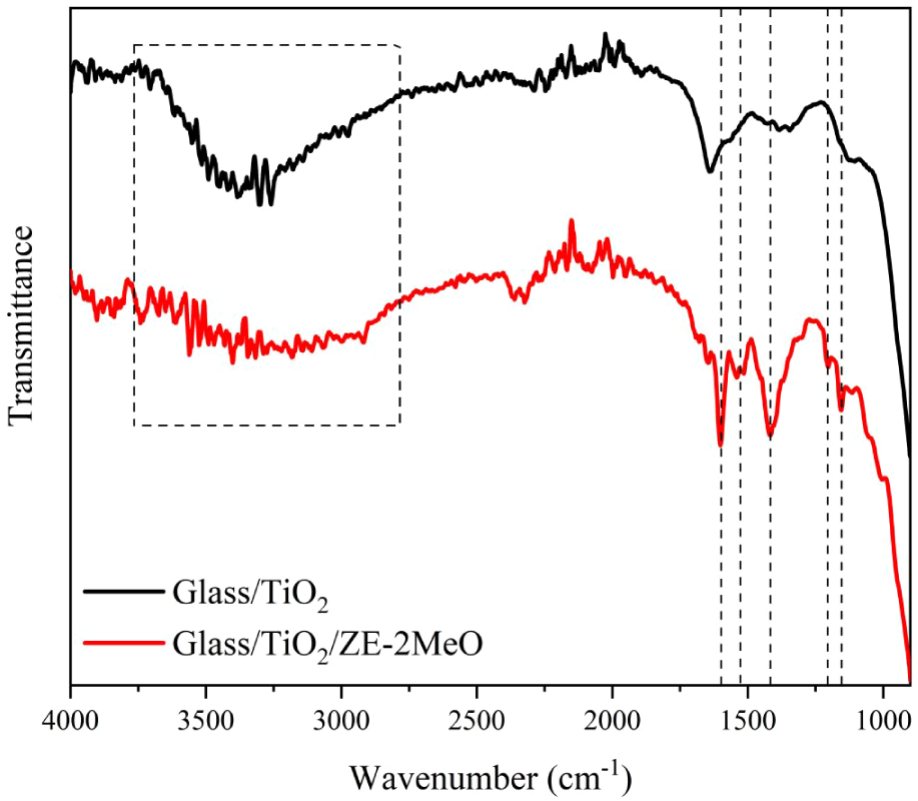
FTIR spectra of bare and ZE-2OMe-modified TiO_2_ films.

XPS analysis provides molecular-level insight into the surface chemistry of the TiO_2_/ZE-2OMe sample. As summarized in Table 2, the O 1s spectrum can be deconvoluted into two main components: the dominant peak at 529.62 eV is assigned to the lattice O^2–^ (Ti–O) of TiO_2_, while the higher-binding-energy component at 530.95 eV is attributed to surface hydroxyl/adsorbed oxygen species (−OH/H_2_O) on the oxide surface (Figure 4a).^38^ The latter contribution is commonly observed for TiO_2_ surfaces and can serve as an interaction site for polar functional groups in the organic overlayer.^39,40^ Consistently, the C 1s spectrum comprises a main hydrocarbon contribution at 284.65 eV assigned to C–C/C=C, together with oxygenated carbon components at 285.71 eV (C–O) and 288.40 eV (O–C=O) (Figure 4b).^41^ The presence of the O–C=O feature is consistent with the carboxylic anchoring group of ZE-2OMe and supports the formation of an organic overlayer on TiO_2_ through carboxylate-based surface interactions. Overall, the combined O 1s and C 1s features confirm the presence of ZE-2OMe-derived functional groups on the TiO_2_ surface and are consistent with successful surface modification.^42^

**Table 2:** Summary of Binding Energy Values Obtained from the High-Resolution XPS Spectra of ZE-2OMe

	O 1s (eV)		C 1s (eV)		
Samples	Ti–O (lattice O_2_^–^)	–OH/adsorbed H_2_O	C–C/C=C	C–O	O=C–O
**ZE-2OMe**	529.62	530.95	284.65	285.71	288.40

**Figure 4:**
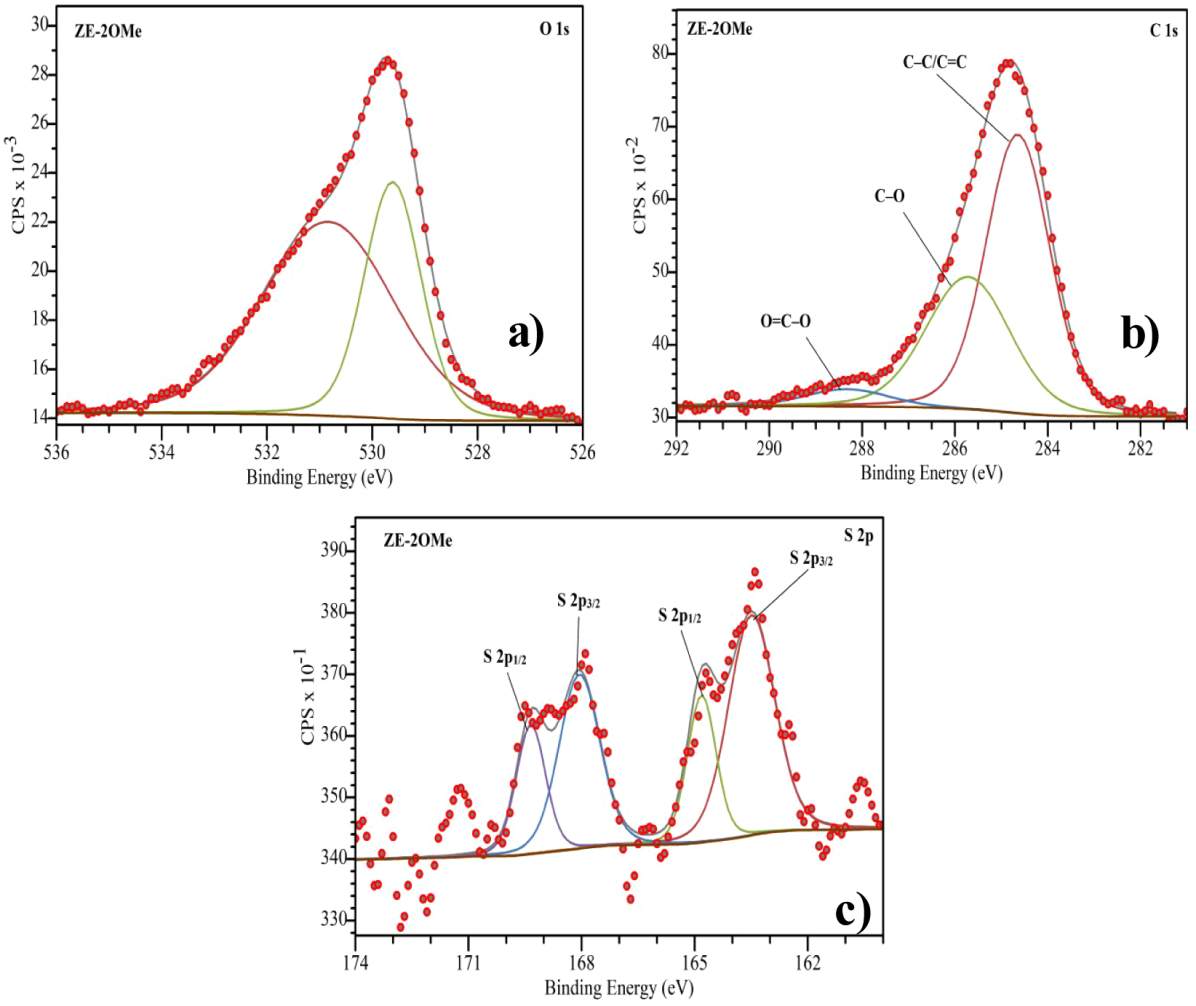
XPS analyses of the TiO_2_/ZE-2OMe thin film: high-resolution spectra of (a) O 1s, (b) C 1s, and (c) S 2p.

Further insight into the sulfur chemical environment of ZE-2OMe was obtained from high-resolution S 2p spectra and peak fitting analysis (Figure 4c). The S 2p envelope was modeled using standard spin–orbit doublets (S 2p_3/2_ and S 2p_1/2_) with a fixed splitting of ∼1.2 eV and an intensity (area) ratio of 2:1, consistent with established XPS protocols. The spectra were acquired on TiO_2_/ZE-2OMe films in the absence of N719; therefore, the S signal originates solely from the coadsorbent layer. In ZE-2OMe, two distinct S 2p doublets were required to reproduce the experimental line shape (Table 3). The lower-binding-energy doublet, with S 2p_3/2_ at 163.49 eV and S 2p_1/2_ at 164.69 eV, is assigned to nonoxidized sulfur in a thiophene-type environment. A second, higher-binding-energy doublet centered at 168.06 eV (S 2p_3/2_) and 169.34 eV (S 2p_1/2_) is attributed to oxidized sulfur contributions, which may arise from minor surface oxidation during sample processing and ambient air exposure and/or from interfacial sulfur species on oxide substrates induced by oxide-driven polarization and charge redistribution.^43–46^ Similar high-BE sulfur components have been reported for thiophene-based organic layers interfaced with TiO_2_ and are commonly discussed within the ∼168–170 eV window characteristic of oxidized organic sulfur environments.^47–49^ Given DMSO-based processing, a contribution from adsorbed sulfoxide-containing solvent cannot be fully excluded.^50,51^ The fitted fwhm values (0.94–1.28 eV) fall within the expected range for organic S 2p components, considering the instrumental energy resolution, signal-to-noise and interfacial heterogeneity, supporting a chemically and physically reasonable fit.^52^ These findings confirm the successful immobilization of ZE-2OMe on the TiO_2_ surface. Such binding characteristics are highly relevant for interfacial engineering in photovoltaic applications like DSSCs. In particular, the ability to passivate potential trap sites on TiO_2_, suppress recombination processes, and improve interfacial charge transfer highlights the critical role of this multisite binding capability.

**Table 3:** Peak Fitting Results of the S 2p XPS Spectra for ZE-2OMe Sample

Spin State	ZE-2OMe	Area	fwhm (eV)	Rel. area (%)
2p_3/2_	163.49	560.8	1.26	40.15
2p_1/2_	164.69	270.5	1.08	16.73
2p_3/2_	168.06	419.0	1.28	28.04
2p_1/2_	169.34	225.3	0.94	15.08

EIS is widely used to probe charge transport, interfacial charge-transfer kinetics, and recombination processes in DSSCs. By applying a small-amplitude AC perturbation over a broad frequency range and measuring the resulting complex impedance response, EIS enables the quantitative extraction of key parameters, including resistive and capacitive elements and the associated characteristic time constants.^53–55^ In DSSCs, Nyquist and Bode diagrams, interpreted through equivalent-circuit modeling, are highly informative for understanding charge transfer and recombination at the TiO_2_/dye/electrolyte interface. The fitted equivalent circuit comprises a series resistance (R_s_) in series with two interfacial RC elements modeled by constant phase elements: (i) the counter-electrode/electrolyte interface, represented by a resistance (R_1_) in parallel with CPE_1_ and (ii) the TiO_2_/dye/electrolyte interface represented by a resistance (R_2_) in parallel with CPE_2_. Here, R_s_ accounts for the overall ohmic losses in the cell (TCO sheet resistance, contacts, and electrolyte). Following the widely adopted DSSC EIS convention, the high-frequency semicircle is operationally assigned to the counter-electrode/electrolyte charge-transfer process (R_1_|CPE_1_), reflecting the electrocatalytic reduction of the redox mediator at the counter electrode. The mid-to-low-frequency semicircle is assigned to the photoanode-side interfacial process (R_2_|CPE_2_), which is commonly associated with interfacial charge accumulation and back-electron recombination of electrons in TiO_2_ with oxidized redox species at the TiO_2_/dye/electrolyte interface.^11,56^ To account for the nonideal (depressed) impedance arcs, the interfacial capacitive contributions were modeled using CPEs. Accordingly, in addition to the resistances, we report the fitted CPE parameters Q and n (Table 4), where n (0 < *n* ≤ 1) quantifies the deviation from an ideal capacitor (*n* = 1). Lower n values indicate a more dispersed interfacial response with distributed time constants. The fitting quality was evaluated using *χ*2 and R^2^ (Table 4); the low *χ*2 (≈10^–5^) together with R^2^ ≈ 0.999, confirm an excellent agreement between the experimental spectra and the fitted impedance curves.

**Table 4:** Equivalent Circuit Parameters Extracted from EIS Analyses Performed under Dark with Respect to Different ZE-2OMe Concentrations in DSSCs

Parameters	Control	0.01 mM	0.02 mM	0.03 mM
**R**_**S**_ (Ω)	27.6	21.8	26.7	27.4
**R**_**1**_ (Ω)	14.8	5.9	7.4	12.6
**Q**_**1**_ (Ω^**–1**^ **s** ^**n**^)	6.53 × 10^–4^	3.05 × 10^–4^	8.45 × 10^–4^	2.78 × 10^–4^
**n**_**1**_	0.592	0.304	0.544	0.996
**R**_**2**_ (Ω)	139.1	70.1	78.5	106.9
**Q**_**2**_ (Ω^**–1**^ **s** ^**n**^)	2.43 × 10^–4^	2.71 × 10^–3^	3.24 × 10^–4^	4.87 × 10^–3^
**n**_**2**_	0.973	0.396	0.950	0.361
**τ**_**e**_ **(ms)**	5.4	8.1	7.4	6.4
**R** ^**2**^	0.999766	0.999899	0.999889	0.999922
*χ* ^2^	3.967135 × 10^–5^	2.712036 × 10^–5^	4.493167 × 10^–5^	2.492485 × 10^–5^

Figure 5a presents Nyquist plots measured under dark conditions for DSSC devices incorporating 0.01, 0.02, and 0.03 mM of the ZE-2OMe coadsorbent. The large semicircles of the reference device indicate high R_1_ and R_2_, whereas the pronounced reduction of both semicircles at 0.01 mM signifies facilitated carrier injection and suppressed recombination. For 0.02 mM and especially 0.03 mM the semicircles broaden, implying that increasing concentration begins to disrupt transport pathways and enhance recombination.^57^ Moreover, as shown in Figure 5b, the electron lifetime (τ_e_), extracted from the characteristic frequency of the Bode phase response, serves as a quantitative indicator of the recombination kinetics.^58^ The reference device exhibits a prominent high-frequency phase peak, whereas the 0.01 mM sample reveals a more distinct and shifted peak toward lower frequencies, indicating prolonged carrier lifetimes and a delayed recombination process. This spectral behavior aligns with enhanced charge transport properties and reduced interfacial losses. For the 0.02 mM and 0.03 mM samples, the Bode phase maximum shifts toward higher frequencies, indicating a shorter characteristic time constant (reduced τ_e_) and thus faster recombination kinetics, consistent with less favorable interfacial transport under higher molecular coverage. The equivalent-circuit parameters summarized in Table 4 quantitatively support these observations. For the reference device, R_1_ = 14.8 Ω, R_2_ = 139.1 Ω, and τ_e_ = 5.4 ms, indicating substantial internal resistance and pronounced interfacial recombination. With 0.01 mM ZE-2OMe, R_1_ = 5.9 Ω, R_2_ = 70.1 Ω, and τ_e_ = 8.1 ms, demonstrating that interfacial engineering improves charge transfer and significantly suppresses recombination. At 0.02 mM, R_1_ and R_2_ increase to 7.1 Ω and 82.5 Ω, respectively, with τ_e_ = 7.4 ms. For 0.03 mM, the EIS parameters deteriorate further (R_1_ = 12.6 Ω, R_2_ = 106.9 Ω; τ_e_ = 6.4 ms), suggesting that excessive concentration induces interfacial disorder and partially blocks transport pathways. Overall, the EIS analysis reveals a clear concentration-dependent impact of ZE-2OMe on DSSC performance. The 0.01 mM concentration provides optimal conditions in terms of both electrical conductivity and interfacial stability, positively influencing carrier dynamics.^57^

**Figure 5:**
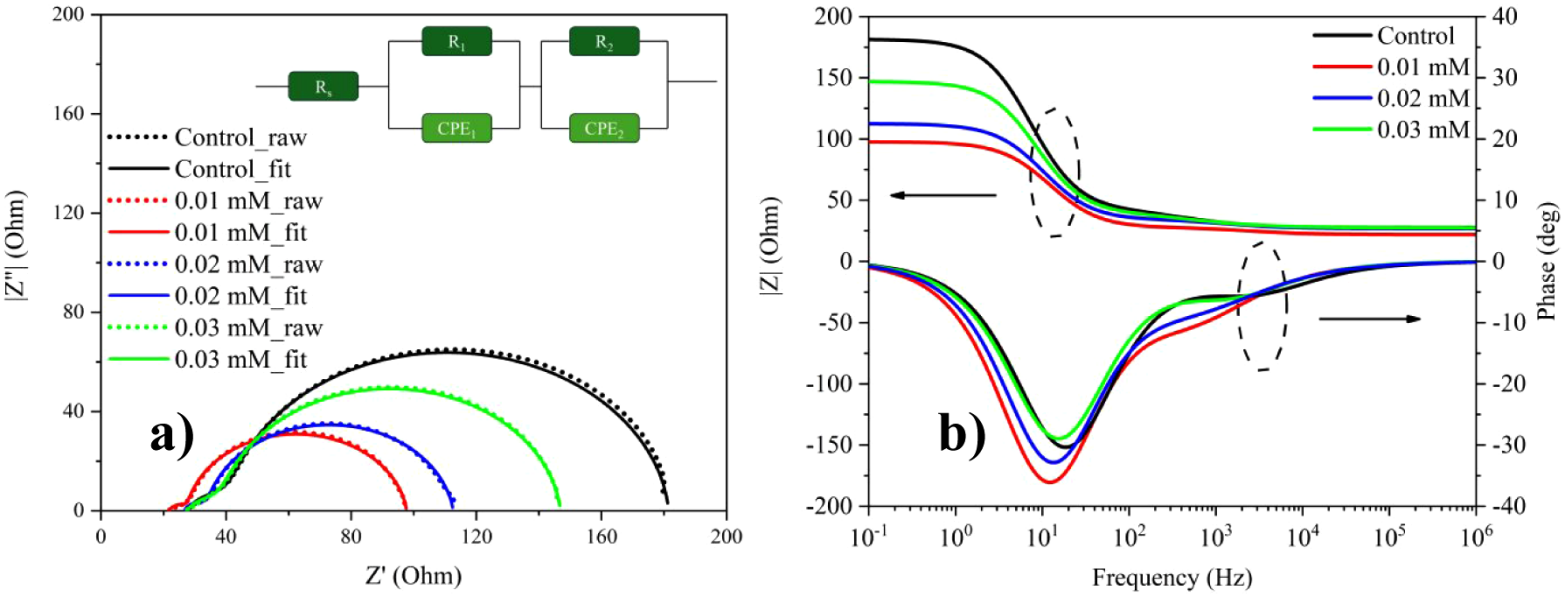
(a) Nyquist plots; (b) Bode plots measured in the dark for DSSCs modified with different concentrations of ZE-2OMe coadsorbent.

Systematic evaluation via J–V and EIS measurements highlights the concentration-dependent role of ZE-2OMe in tuning interfacial charge transfer, recombination suppression, and overall device efficiency. Compared to the reference device (*J*_sc_ = 10.6 mAcm^2^, *V*_oc_ = 655 mV, FF = 50%, PCE = 3.5%), the device modified with 0.01 mM of ZE-2OMe exhibits a remarkable improvement across all photovoltaic parameters, achieving a *J*_sc_ of 12.6 mA/cm^2^, *V*_oc_ of 685 mV, FF of 63%, and a PCE of 5.4% (Figure 6a). This improvement is attributed to effective surface passivation on TiO_2_ via controlled coadsorbent adsorption and optimized interfacial dipole alignment. These observations are quantitatively supported by the EIS analysis: in the device with 0.01 mM ZE-2OMe, R_s_ = 21.8 Ω, R_1_ = 5.9 Ω, R_2_ = 70.1 Ω, and τ_e_ = 8.1 ms, indicating more efficient charge transfer with reduced interfacial resistances and suppressed recombination. By contrast, the control device shows R_1_ = 14.8 Ω, R_2_ = 139.1 Ω, and τ_e_ = 5.4 ms, consistent with recombination-dominated behavior and more limited charge transport. As the ZE-2OMe concentration is increased further, particularly for 0.02 mM and 0.03 mM, both J–V and EIS data indicate a decrease in performance. At 0.02 mM, *J*_sc_ = 10.4 mA/cm^2^, *V*_oc_ = 680 mV, FF = 60.5%, and PCE = 4.3%; at 0.03 mM, *J*_sc_ = 10.3 mA/cm^2^, *V*_oc_ = 660 mV, FF = 56.9%, and PCE = 3.9%. Consistently, the EIS parameters increase again at these higher concentrations, reaching R_1_ = 12.6 Ω, R_2_ = 106.9 Ω, and τ_e_ = 6.4 ms for 0.03 mM, which suggests that excessive coadsorbent coverage induces interfacial disorder, disrupts transport pathways, and increases the tendency for recombination. A concentration of 0.01 mM yields the optimal balance across optical, chemical, and electrochemical parameters, underscoring how molecular coadsorbent integration can decisively determine DSSC efficiency. Additionally, the device-to-device reproducibility was evaluated by measuring five independently fabricated cells for control and ZE-2OMe (0.01 mM)-based cells. The averaged J–V parameters are summarized in Table S1. In five independently fabricated devices, the control cells exhibited average performance of *J*_sc_ = 9.88 mA/cm^2^, *V*_oc_ = 656 mV, FF = 52.92%, and PCE = 3.36%. In contrast, the ZE-2OMe devices showed higher average values (*J*_sc_ = 12.88 mA/cm^2^, *V*_oc_ = 681 mV, FF = 61.98%, and PCE = 5.26%), confirming that the performance improvement is reproducible.

**Figure 6:**
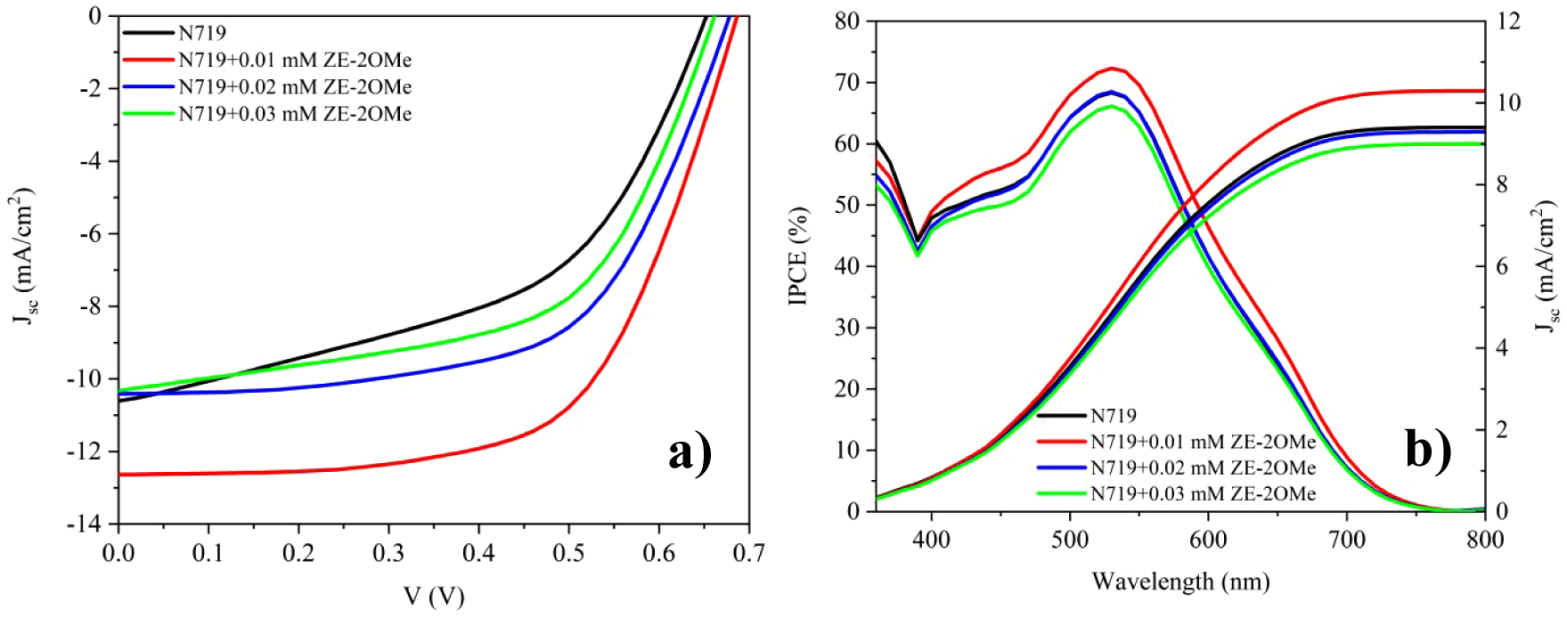
(a) J–V characteristics and (b) IPCE graphs of DSSCs obtained by applying the ZE-2OMe coadsorbent at different concentrations.

Importantly, the IPCE spectra do not exhibit any additional feature or enhancement in the 390–410 nm region where ZE-2OMe absorbs; instead, the main IPCE changes occur across the N719-dominated visible range (≈450–650 nm), indicating that the *J*_sc_ improvement is primarily driven by interfacial/electronic effects rather than complementary light harvesting. Accordingly, the photocurrent gain is attributed to improved charge collection and suppressed interfacial losses. This interpretation is consistent with the EIS results, which show reduced interfacial resistive losses and a prolonged electron lifetime at the optimal ZE-2OMe concentration, implying slower back-electron transfer at the TiO_2_/dye/electrolyte interface.^59^ Such recombination suppression rationalizes the concurrent increase in *V*_oc_, while the reduced resistive losses near the maximum-power point account for the higher FF.^13^ A slight difference was observed between the *J*_sc_ values obtained from the J–V curves and those calculated by integrating the IPCE spectra (Figure 6b and Table 5).

**Table 5:** J–V Characteristics Measured under Illumination for DSSC Devices Prepared with Different ZE-2OMe Concentration

ZE-2OMe Conc.	N719 Conc.	J_sc_ (mA/cm^2^)^J‑V^	J_sc_ (mA/cm^2^)^IPCE^	V_oc_ (mV)	FF (%)	PCE (%)
**0**	0.5 mM	10.6	9.4	655	50.0	3.5
**0.01 mM**	**0.5 mM**	12.6	10.4	685	63.0	5.4
**0.02 mM**	**0.5 mM**	10.4	9.3	680	60.5	4.3
**0.03 mM**	**0.5 mM**	10.3	9.0	660	56.9	3.9

Such discrepancies are common in photovoltaic devices and stem from several fundamental causes. First, IPCE is measured over a finite spectral window, whereas J–V is recorded under broad-spectrum simulated sunlight. Because the IPCE measurement range may not fully cover the solar spectrum, the integrated *J*_sc_ can be underestimated. Moreover, the monochromatic excitation employed in IPCE measurements can induce carrier transport and recombination behavior that differs from that under the broadband, steady-state illumination used for J–V characterization. Second, the lower irradiation typically used in IPCE can limit the transport and regeneration processes within the device, resulting in apparent lower carrier collection efficiency. In addition, systematic errors such as IPCE system calibration, optical losses in fiber delivery, monochromator throughput, and detector responsivity may further depress the integrated *J*_sc_. By contrast, J–V curves more closely represent real operating conditions under the full spectrum and thus often yield higher *J*_sc_ values. In this study, the differences between the two methods are within acceptable limits and are commonly reported in the literature to be on the order of 10–20%. In summary, the observed deviations arise from intrinsic limitations of the measurement techniques and do not materially affect the overall photovoltaic assessment of the devices.^60,61^

## Conclusion

In this work, the impact of applying ZE-2OMe at different concentrations to the TiO_2_ surface on DSSC performance was comprehensively evaluated. XPS and FTIR analyses demonstrated chemical anchoring of ZE-2OMe to the surface and the formation of an interface favorable for electron transfer. TRPL and steady-state PL characterizations demonstrated that the incorporation of 0.01 mM ZE-2OMe yields the most favorable optoelectronic balance among the tested concentrations. The corresponding *τ* extends to 4.87 ns, substantially exceeding that of the pristine reference (2.84 ns). According to EIS, R_1_ = 5.9 Ω and R_2_ = 70.1 Ω at 0.01 mM reach their minimal values among the tested conditions, pointing to optimal interfacial stability. All characterization results are directly corroborated by J–V measurements: *J*_sc_ = 12.6 mA/cm^2^, *V*_oc_ = 685 mV, FF = 63.0%, and PCE = 5.4% at 0.01 mM, clearly identifying this concentration as optimal for device performance. At higher coadsorbent concentrations, PL lifetimes and device efficiency both decrease, indicating that excessive molecular adsorption restricts transport pathways and adversely affects efficiency. Consistently, dye-loading measurements show that ZE-2OMe does not increase N719 loading, further supporting that the improved *J*_sc_ and overall efficiency originate from reduced interfacial losses and optimized charge-transfer/recombination dynamics at the TiO_2_/dye/electrolyte interface. Overall, not only the chemical structure but also the concentration of ZE-2OMe critically determines DSSC performance. This study highlights that optimizing the coadsorbent ratio in SAM engineering is essential for simultaneously improving interfacial passivation and transport processes.

## Supplementary Material



## Data Availability

All data supporting the findings of this study are available in the main manuscript and its Supporting Information.

## Notes

^1^H NMR, ^13^C NMR, TOF-MS, and FTIR spectra of the synthesized molecules and N719/TiO_2_/FTO films; thicknesses of the mesoporous TiO_2_ (m-TiO_2_) and compact TiO_2_ (c-TiO_2_) layers; and the detailed synthetic procedure for ZE-2OMe (PDF)
