# Supplementary material for: Concentration-Dependent Interfacial Engineering with a ZE-2OMe Co-adsorbent for Enhanced DSSC Performance
Source: ACS Omega. 2026 May 6;11(19):28381–90. doi: 10.1021/acsomega.6c00348 (PMC13191510; doi:10.1021/acsomega.6c00348)
Supplement: Supplementary file 1 [file ao6c00348_si_001.pdf]

Supporting Information

Concentration-Dependent Interfacial Engineering with a ZE-2OMe Co-Adsorbent for Enhanced DSSC Performance

[1,2]Necip Ali Tuna, [3]Mesude Zeliha Arkan, [4]Mustafa Can,[2,]Adem Mutlu,[4,*]Cem Tozlu*

[1]Department of Materials Science and Engineering, Izmir Katip Celebi University, Izmir, Türkiye

[2]Ege University, Solar Energy Institute, 35100, Izmir, Türkiye

[3]Institute of Chemistry, University of Silesia in Katowice, Szkolna 9, Katowice 40-006, Poland

[4]Graphene Application and Research Center, Izmir Katip Celebi University, Cigli, 35620 Izmir, Türkiye

***Corresponding authors E-mail**: cem.tozlu@ikcu.edu.tr, adem.mutlu@ege.edu.tr*

This file includes:

Figure S1 to S10

Table S1

Synthesis of 4- [5 '- (3,5-dimethoxyphenyl) -2,2- bithiophen -5-yl] benzoic acid (ZE-2OMe): All chemicals, reagents, and solvents were obtained from standard commercial suppliers and employed directly without any additional purification steps. Prior to use, the glassware was thoroughly dried in an oven, and every reaction was conducted under an inert nitrogen (N_2) atmosphere.

Synthesis of Methyl 4-(5'-phenyl[2,2'-bithiophen]-5-yl) benzoate: In a Schlenk flask, methyl-4-iodobenzoate (200 mg, 0.53 mmol) and 2-(5′-bromo-2,2′-bithien-5-yl)-4,4,5,5-tetramethyl-1,3,2-dioxaborolane (96.3 mg, 0.79 mmol) were dissolved in 1,2-dimethoxyethane (DME, 20 mL). After the reaction temperature reached 50 °C, Pd(dppf)Cl_2 and K_2CO_3 were introduced, and the mixture was then refluxed under a nitrogen atmosphere overnight. Reaction progress was followed by thin-layer chromatography (TLC) until completion. Upon completion, the reaction mixture was partitioned between dichloromethane (CH_2Cl_2) and water using equal volumes. The organic phase was concentrated under reduced pressure using a rotary evaporator to give the crude residue, which was subsequently purified by silica gel column chromatography (SiO_2; CH_2Cl_2/n-hexane = 1:1, v/v) to afford the target compound as a yellow powder. ^{1}H NMR (100 MHz, DMSO): δ 7.99 (d, 2H), 7.85–7.80 (m, 2H), 7.40 (t, 1H), 7.25 (s, 2H), 3.86 (s, 3H).

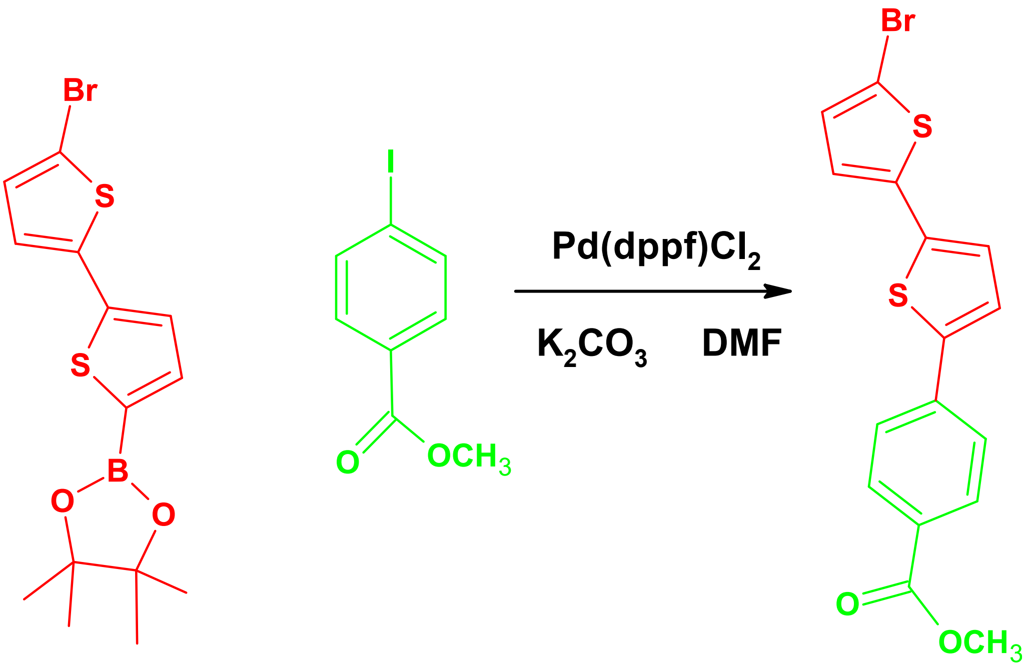

Figure S1. Synthetic route for the synthesis of Synthesis of Methyl 4-(5'-phenyl[2,2'-bithiophen]-5-yl) benzoate.

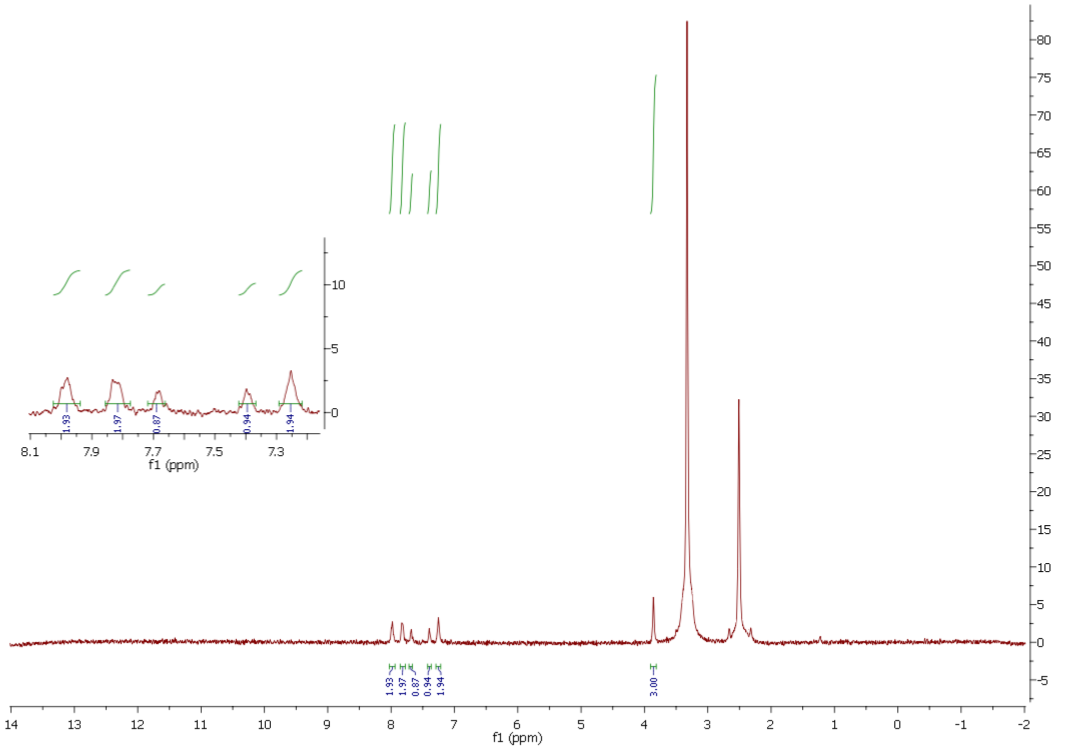

Figure S2. ^{1}H-NMR spectrum of Methyl 4-(5'-phenyl[2,2'-bithiophen]-5-yl) benzoate

4- [5 '- (3,5-dimethoxyphenyl) -2,2- bithiophen -5-yl] benzoic acid (ZE-2OMe): In a Schlenk flask, 4-(5′-bromo-2,2′-bithiophen-5-yl) benzoate (200 mg, 0.53 mmol) and 3,5-trimethoxybenzeneboronic acid (143.8 mg, 0.79 mmol) were dissolved in 1,2-dimethoxyethane (DME, 20 mL). Once the mixture was heated to 50 °C, Pd(dppf)Cl_2 (22 mg, 0.03 mmol) and K_2CO_3 (1 M, 1 mL) were added. The reaction was then refluxed under a nitrogen atmosphere overnight, and its progress was tracked by thin-layer chromatography (TLC) until completion. After cooling, the mixture was extracted with equal volumes of CH_2Cl_2 and water. The organic layer was concentrated under reduced pressure using a rotary evaporator to yield the crude product, which was purified by silica gel column chromatography (SiO_2; CH_2Cl_2/n-hexane = 1:1, v/v) to obtain a yellow powder. For the subsequent hydrolysis, the purified intermediate was dissolved in a 1:1 (v/v) THF/ethanol mixture in a round-bottom flask. KOH (1 M, 0.5 mL) was added, and the reaction was refluxed overnight. The solvents were removed under reduced pressure, after which deionized water (10 mL) was introduced and the solution was acidified with 1 M HCl to pH 3–4. The yellow product precipitated, was collected by filtration, washed thoroughly with water, and dried overnight. ^{1}H NMR (100 MHz, DMSO): δ 7.96 (d, 2H), 7.79 (d, 2H), 7.67 (s, 1H), 7.56 (s, 1H), 7.41 (s, 2H), 6.80 (s, 2H), 6.48 (s, 1H), 3.80 (s, 6H). ^{13}C NMR (100 MHz, DMSO): δ 166.96, 161.53, 142.94, 141.22, 137.65, 135.82, 135.33, 130.68, 129.94, 126.81, 125.97, 125.43, 55.93.

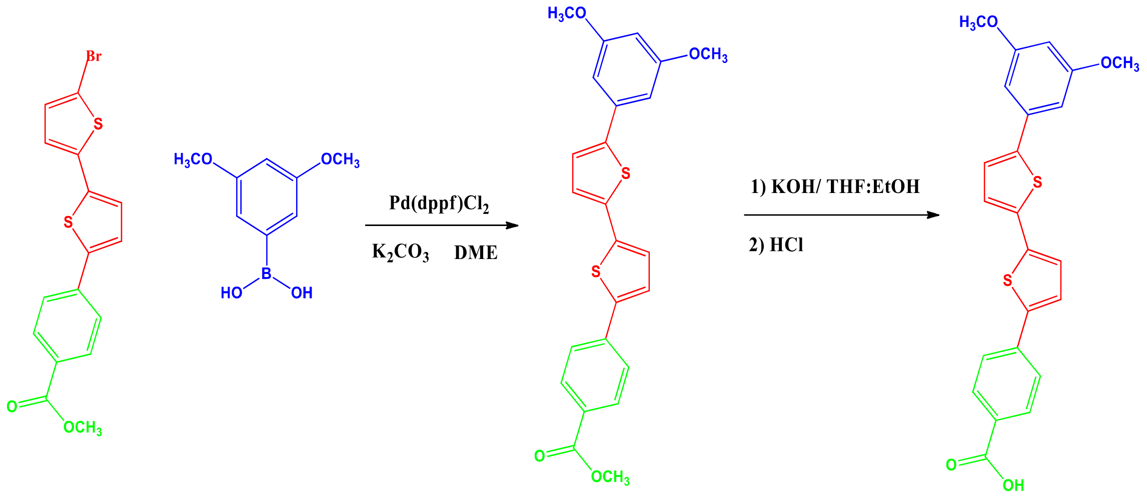

Figure S3. Synthetic route for the synthesis of ZE-2OMe.

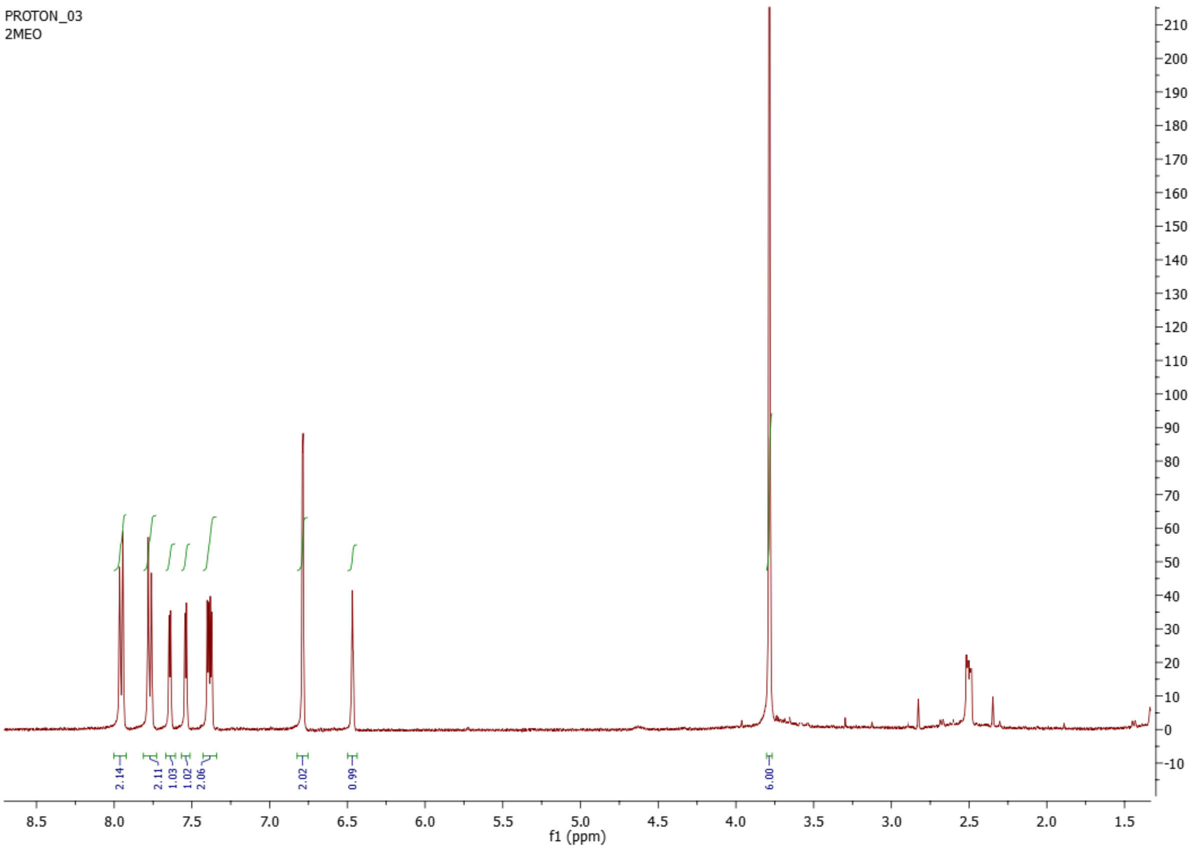

Figure S4. ^{1}H-NMR spectrum of ZE-2OMe.

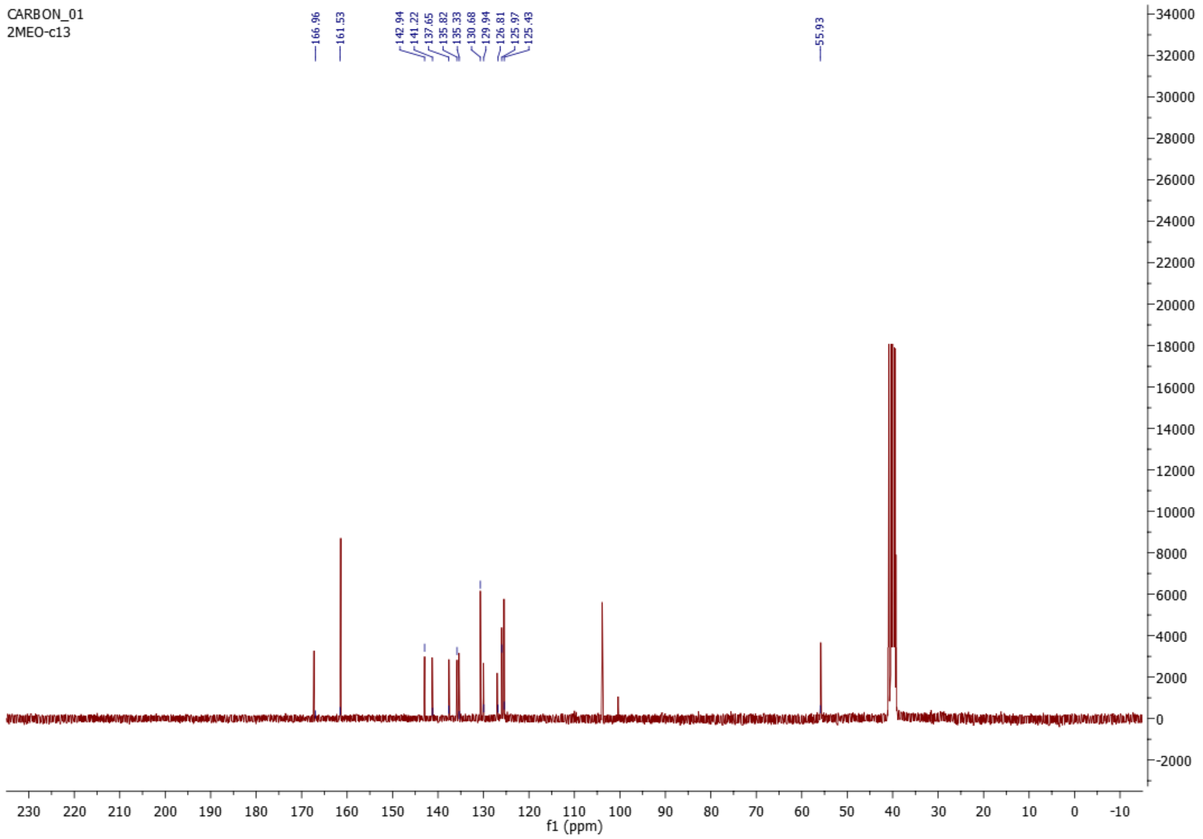

Figure S5. ^{13}C-NMR spectrum of ZE-2OMe.

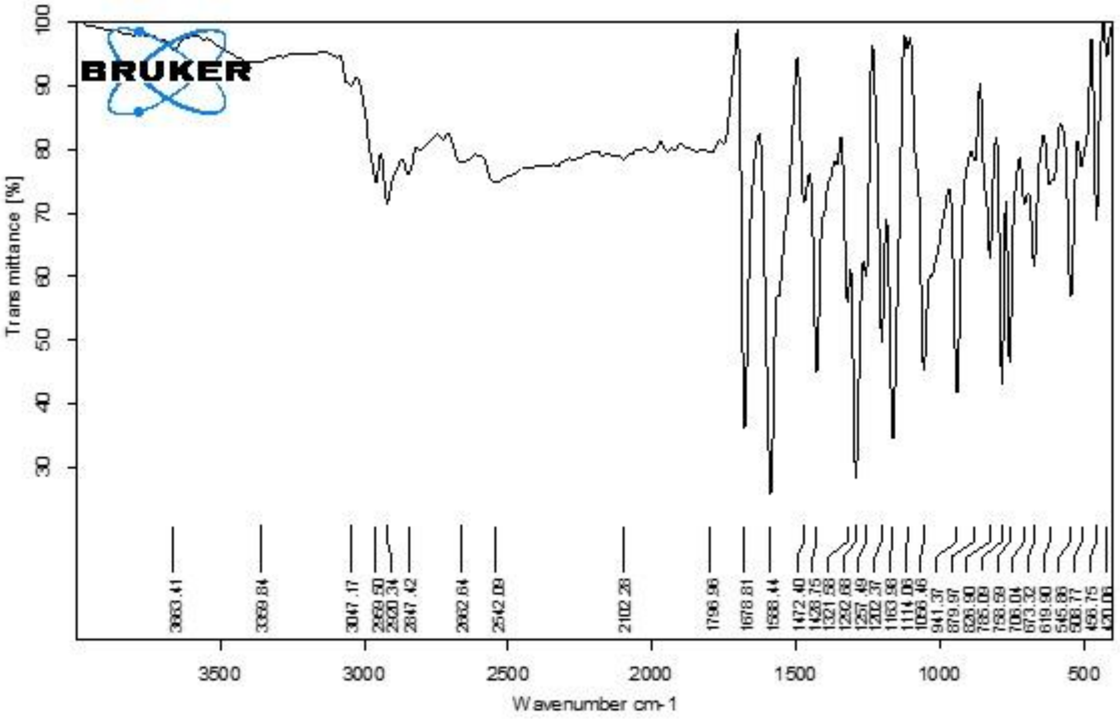

Figure S6. FTIR spectrum of ZE-2OMe.

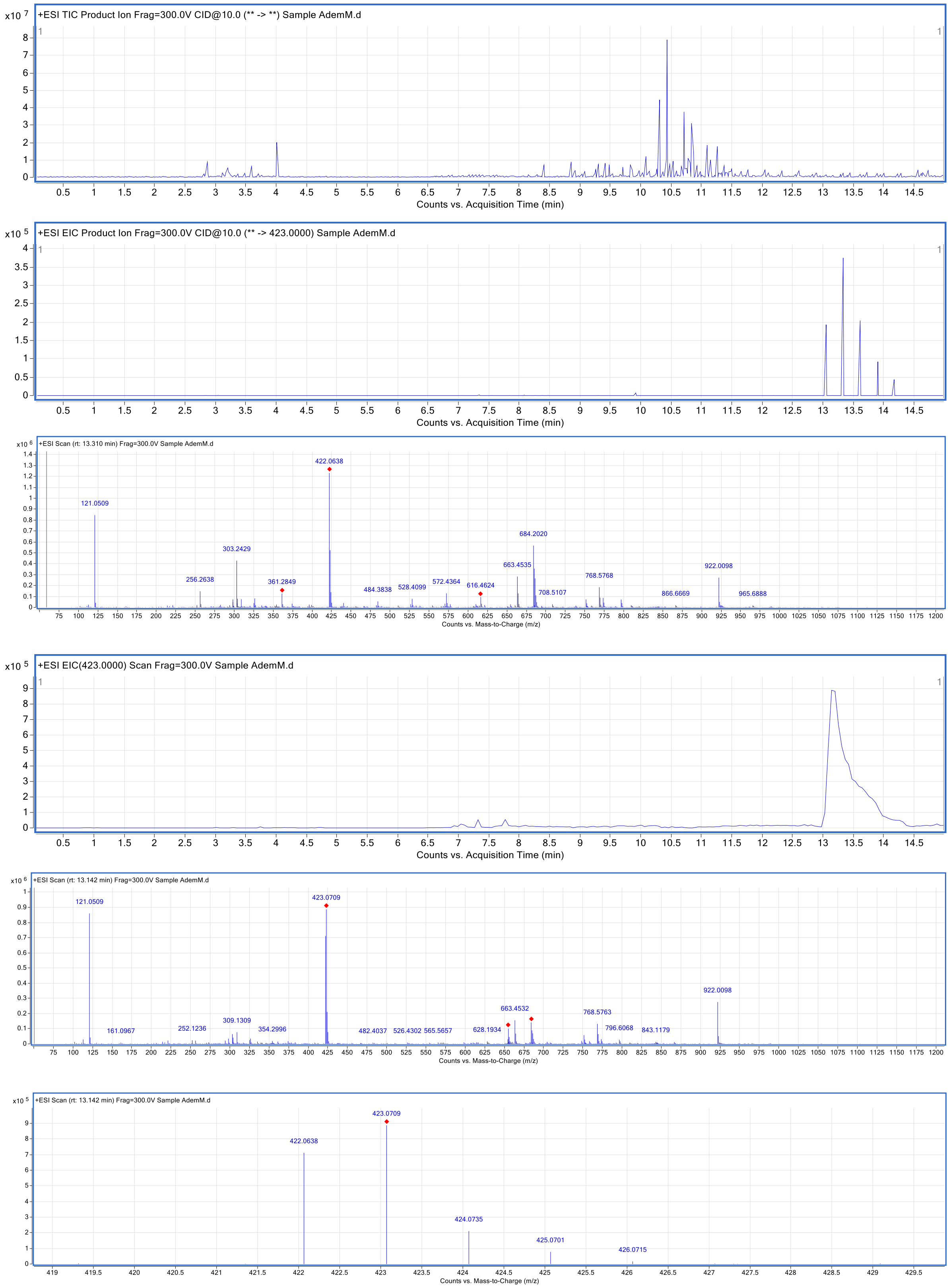

Figure S7. Positive ESI-TOF-MS characterization of ZE-2OMe. The TIC, EIC extracted for *m/z* 423, full-scan mass spectrum, and MS/MS fragmentation spectrum of the selected precursor ion are presented. The detected ion at *m/z* 423 is attributed to the $[M+H]^+$ protonated adduct of ZE-2OMe.

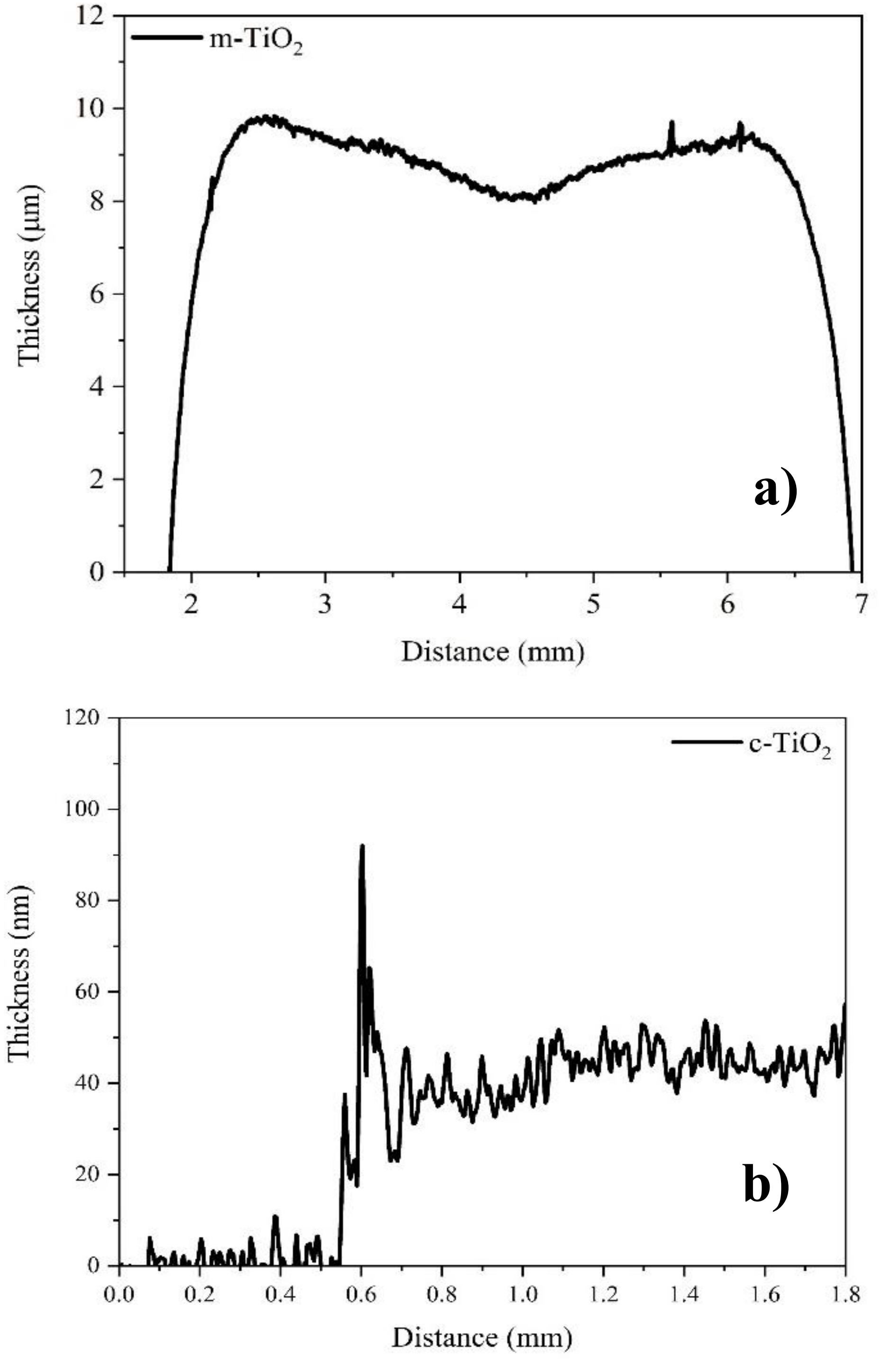

Figure S8. Thickness profile of the a) m-TiO_2 and b) c-TiO_2 layer.

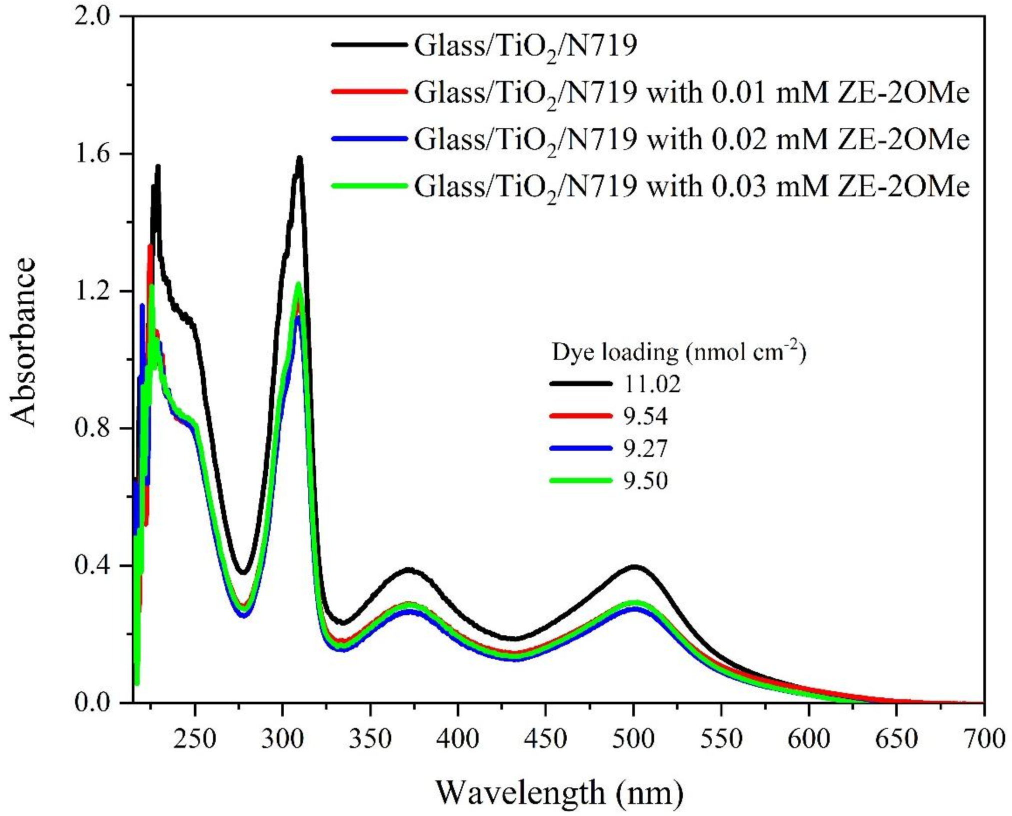

Figure S9. UV–Vis absorption spectra of N719 desorbed from TiO_2 photoanodes.

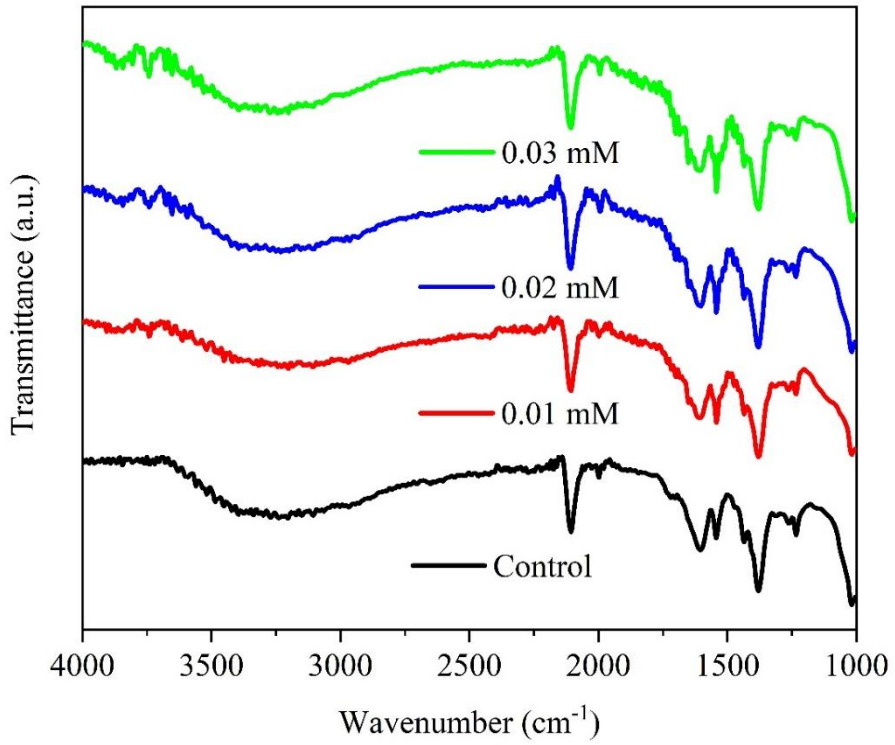

Figure S10. FTIR spectra of N719/TiO_2/FTO films modified by adding the ZE-2OMe co-adsorbent at different concentration.

Table S1. Averaged J–V characteristics of DSSCs fabricated with and without ZE-2OMe.

ID	J_{sc} (mA/cm^2)	V_{oc} (mV)	FF (%)	PCE (%)
**N719**	10.1	660	50.8	3.4
	10.6	660	48.8	3.4
	10.7	640	48.9	3.3
	10.6	655	50.0	3.5
	9.7	660	54.2	3.4
**Average**	9.88	656	52.92	3.36
**N719+0.01 mM ZE-2OMe**	12.6	685	63.0	5.4
	14.5	680	61.4	5.2
	12.7	680	61.5	5.3
	12.5	680	62.7	5.4
	12.1	680	61.3	5.0
**Average**	12.88	681	61.98	5.26
